# A new type of biomarker for heat stress: insights from immunology

**DOI:** 10.3389/fimmu.2026.1742202

**Published:** 2026-02-12

**Authors:** Chunmei Ye, Zhipeng Zhang, Zhangping Yang

**Affiliations:** 1 College of Animal Science and Technology, Yangzhou University, Yangzhou, China; 2 Experimental Farm, Yangzhou University, Yangzhou, China; 3 Zhejiang Key Laboratory of Cow Genetic Improvement and Milk Quality Research, Wenzhou, China

**Keywords:** dairy cow, gut-mammary axis, heat stress, immune biomarkers, thermotolerance

## Abstract

Global warming exacerbates heat stress in dairy cows, while traditional indicators like the Temperature-Humidity Index (THI) struggle to accurately reflect individual physiological response variations. This review systematically analyzes the dynamic changes in the bovine immune system under heat stress and proposes immune-related biomarkers as a novel strategy for early warning and individualized assessment. Research reveals that heat stress compromises the innate immune barrier function of cows, suppressing neutrophil chemotaxis and antimicrobial peptide synthesis, leading to a decline in the opsonophagocytic index and endotoxin translocation. Expression of intestinal tight junction proteins (e.g., Claudin-1, Occludin) is significantly downregulated (by ~40%), causing endotoxin translocation (serum LPS increases ~3-fold) and disruption of the blood-milk barrier, increasing mastitis risk by 2–3 times. Regarding adaptive immunity, the proliferation and differentiation of T/B cells are impaired, the CD4+/CD8+ T cell ratio decreases, antibody affinity maturation is suppressed, and the efficiency of immune memory formation post-vaccination is reduced. Concerning mammary immunity, the number of viable granulocytes in milk decreases, mammary epithelial cells increase, pro-inflammatory cytokine levels rise, and the risk of clinical mastitis increases 2–3 fold. Furthermore, heat stress induces metabolic reprogramming, gut microbiota dysbiosis, oxidative stress, and activation of the hypothalamic-pituitary-adrenal (HPA) axis. Novel biomarkers, such as Heat Shock Proteins (HSPs), Neutrophil-to-Lymphocyte Ratio (NLR), cytokine profiles (e.g., IL-6/TNF-α), acute-phase proteins (e.g., Haptoglobin, Hp), and epigenetic markers, provide crucial targets for heat-tolerant breeding and early intervention, promising to enhance the climate resilience of the dairy industry.

## 1 Introduction

The stark reality of global warming poses unprecedented systemic challenges to animal husbandry, particularly highly intensive dairy farming (1). According to authoritative data from the Intergovernmental Panel on Climate Change (IPCC), the current global average temperature has risen by approximately 1°C compared to pre-industrial levels. This seemingly modest increase has directly led to a significant rise in the frequency, intensity, and duration of extreme heatwave events (2, 3). Dairy cows, as core production animals, exhibit heightened sensitivity to high-temperature environments due to their unique physiology, large body size, thick hide (hindering heat dissipation), and relatively underdeveloped sweat gland system (relying primarily on respiratory cooling) (4). When environmental temperatures persistently exceed their thermoneutral zone (TNZ, typically considered between 5 °C and 25 °C), the cow’s thermoregulatory mechanisms become overwhelmed, rapidly triggering the Heat Stress Response (HSR) (5).

**Figure 1:**
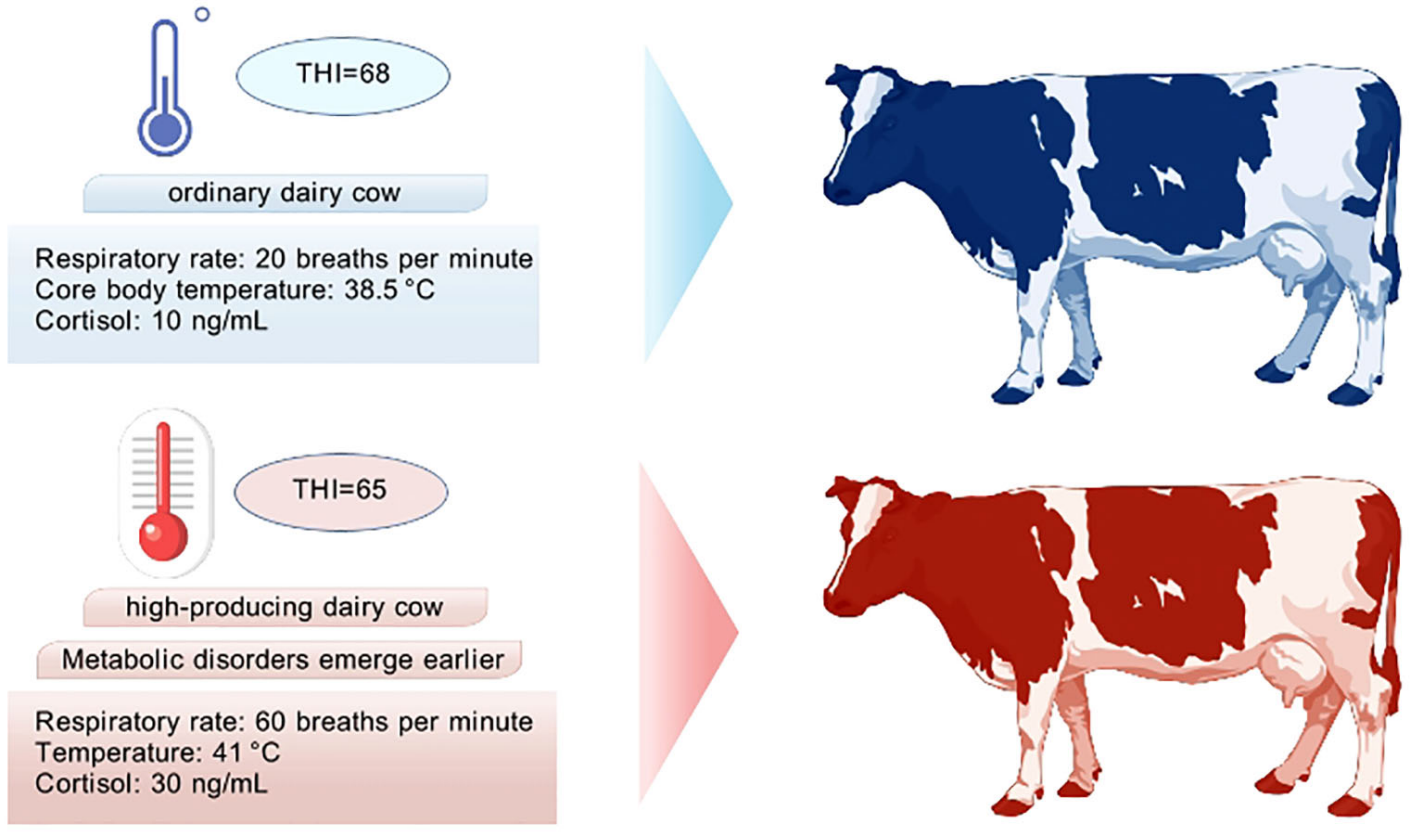
Heat-stress triggers and individual threshold differences. Individualized heat-stress threshold variation. While a THI of 68 is conventionally used as the population-level threshold, high-yielding cows exhibit significant physiological disruption at THI 65, indicating that immune biomarkers should be incorporated for early warning.

Heat stress is far more than mere discomfort; it initiates a cascade of complex physiological, metabolic, and immune dysfunctions, ultimately translating into substantial economic and production losses (6). Long-term assessment of dairy cow heat stress has primarily relied on traditional metrics like rectal temperature measurement and the Temperature-Humidity Index (THI) (7). While a THI >68 is widely regarded as the critical threshold for heat stress, this approach has significant limitations: it is inherently an environmental parameter incapable of precisely capturing individual variations in physiological responses to heat stress (8). For instance, high-yielding cows, due to greater metabolic heat production, may exhibit significant metabolic disturbances (e.g., exacerbated oxidative stress, negative energy balance) and physiological changes (e.g., sharply elevated respiratory rate, increased core body temperature) at an environmental THI as low as 65, well before the average herd threshold is reached (**Figure 1**) (9).

Consequently, the development and application of novel biomarkers capable of enabling early warning, possessing high specificity, and accurately reflecting the internal physiological state of the organism have become a forefront hotspot and urgent need in current dairy heat stress research. In this exploration, the immune system, serving as the body’s first line of defense and central regulator against external environmental stimuli (including heat stress), exhibits dynamic activation states intricately linked to the onset, progression, and consequences of heat stress (10–12). Changes in immune-related indicators (e.g., quantity and activity of specific immune cell subsets, cytokine profiles, acute-phase proteins, immune-related gene expression profiles) often precede or accompany noticeable declines in production performance and the emergence of clinical symptoms, providing a valuable window for early intervention (13–15).

The core objective of this review is to systematically focus on the complex and dynamic changes occurring in the bovine immune system under heat stress conditions. By synthesizing the latest research advances, we will elaborate on the discovery pathways, validation processes, and application potential in research and practice for a range of novel immune-related biomarkers (e.g., specific inflammatory cytokines, heat shock proteins, immune cell functional indicators, epigenetic markers). These markers not only facilitate more precise and individualized assessment of a cow’s heat stress status and tolerance capacity but, more importantly, provide a solid scientific foundation and powerful technical support for breeding new dairy cattle varieties with enhanced thermotolerance (molecular breeding), ultimately bolstering the resilience and sustainability of the dairy industry in the face of global warming.

## 2 Overview of the bovine immune system

### 2.1 Innate immunity

The innate immune system serves as the primary barrier of the cow’s immune defense mechanism, activating rapidly within hours of pathogen invasion. It comprises key components including physical barriers, humoral factors, and immune cells, which collaborate to mount a swift response against invading pathogens (16).

Physical barriers constitute the outermost line of innate defense, encompassing the skin and mucous membranes. The skin’s keratinized layer blocks pathogen entry, while mucous membranes of the respiratory, gastrointestinal, and urogenital tracts clear pathogens via ciliary movement and mucus secretion (17). For example, relaxation time of the teat sphincter increases under heat stress, elevating mastitis risk (18). Humoral factors also play vital roles in innate immunity. Lysozyme in serum disrupts bacterial cell walls, lactoferrin inhibits bacterial proliferation by chelating iron ions, and the complement system lyses pathogens via classical and alternative pathways (19, 20). These humoral factors not only directly kill pathogens but also enhance the phagocytic activity of immune cells (21). Phagocytic cells are core components of innate immunity, including neutrophils and macrophages (22). Neutrophils constitute 40-50% of blood leukocytes and rapidly migrate to infection sites via chemotaxis to phagocytose and kill pathogens (23). Heat stress can reduce neutrophil chemotactic capacity by 30%, impairing their pathogen clearance efficiency (24). Macrophages clear pathogens via phagocytosis and participate in antigen presentation, activating subsequent adaptive immune responses (25).

Through mechanisms involving physical barriers, humoral factors, and immune cells, the bovine innate immune system rapidly responds to pathogen invasion, reducing infection and activating adaptive immunity (26). In production settings, an efficient innate immune system significantly lowers disease incidence in cows, prevents ailments like mastitis, reduces veterinary drug use, improves production performance and milk quality, and increases economic returns (27). However, factors like heat stress can weaken its defensive functions, increasing disease risk.

### 2.2 Adaptive immunity

The bovine adaptive immune system is a crucial component of its immune defense, characterized by high specificity and memory, primarily mediated by B cells and T cells (28). It plays a key role in cow health and productivity, effectively recognizing and eliminating pathogens while providing long-term immune protection (29).

B cells are primarily responsible for humoral immune responses in adaptive immunity, producing specific antibodies to recognize and neutralize pathogens (30). Antibodies (immunoglobulins) are products of B cells and exist in various types, including IgM, IgG, IgA, IgD, and IgE (31). In cows, IgG1 and IgG2 are predominant types, playing significant roles in neutralizing viruses and toxins, and in bacterial agglutination and opsonization (32). IgA primarily functions at mucosal sites like the respiratory and gastrointestinal tracts, preventing pathogen adhesion and invasion (33). T cells are mainly responsible for cell-mediated immune responses, including cytotoxic T cells (CTLs) and helper T cells (Th cells) (34). CTLs directly kill pathogen-infected cells, while Th cells regulate the activity of other immune cells by secreting cytokines (30). In cattle, γδ T cells also play important roles, particularly in neonates, where they may compensate for the immaturity of functions like neutrophils (35).

The adaptive immune system is vital for bovine health and productivity. An efficient adaptive immune system significantly reduces disease incidence, effectively prevents common diseases like mastitis, thereby decreasing veterinary drug use frequency, and subsequently enhances cow productivity and milk quality. For instance, vaccination can induce adaptive immune responses, establishing immune memory and enhancing resistance to specific pathogens (36). Moreover, the long-term persistence of immune memory cells provides sustained protection for cows.

## 3 Impact of heat stress on bovine innate immunity

### 3.1 Impact of heat stress on bovine immune barriers

You may insert up to 5 heading levels into your manuscript as can be seen in “Styles” tab of this template. These formatting styles are meant as a guide, as long as the heading levels are clear, Frontiers style will be applied during typesetting. The bovine immune barrier comprises multi-layered defense mechanisms including the skin barrier, respiratory barrier, and intestinal barrier, which play crucial roles in defending against pathogen invasion and maintaining overall health (37). Heat stress, as a common environmental stressor, can compromise the integrity of these immune barriers through various mechanisms, significantly reducing the cow’s disease defense capacity (38).

The skin, as the first line of immune defense, effectively blocks pathogen entry under normal physiological conditions (39). Under heat stress, cows dissipate heat through sweating and increased respiratory rate; this process can disrupt skin barrier integrity, leading to increased water loss from the skin surface, dryness, and impaired barrier function (40). High temperature and humidity environments alter the skin surface microbiome, increasing the abundance of pathogenic bacteria (e.g., Staphylococcus aureus) and the probability of penetration through the damaged stratum corneum (41, 42). This decline in barrier function facilitates easier pathogen penetration into the body. Prolonged exposure to high temperatures may alter the structure of the skin surface microbial community, significantly increasing the risk of bacterial or fungal infections, further weakening the skin’s defensive capabilities (41). Heat stress exacerbates rumen epithelial sloughing, disrupting its physical barrier, and interferes with pathways related to DNA replication/repair and amino acid metabolism, though no specific impact on tight junction protein or TLR4 signaling expression was found (43). Heat stress may maintain rumen epithelial barrier integrity by upregulating HSP expression, and increased amino acid metabolism in the rumen could affect systemic nutrient utilization (44).

The respiratory immune barrier is also significantly affected by heat stress. High temperatures cause a marked increase in respiratory rate and depth in cows, a physiological cooling mechanism, but frequent respiration also increases opportunities for pathogens to enter the respiratory tract (45, 46). Simultaneously, microbial concentrations in the air may rise under high-temperature conditions, further exacerbating the risk of respiratory infections (47). Studies show that drying mucosa develops micro-fissures, making it easier for airborne pathogenic microbes (e.g., Mycoplasma, Streptococcus pneumoniae) to adhere and invade, thereby increasing clinical respiratory infection rates (48). The respiratory mucosa is a vital barrier against pathogen invasion; its surface mucus layer and ciliary motion effectively clear inhaled pathogens (49). Heat stress can cause drying of the respiratory mucosa and reduced ciliary motility, weakening its defense capacity and increasing the incidence of respiratory infections (50). Research indicates heat stress forces cows to double their respiratory rate (from 20-30/min to 60-80/min), accelerating water evaporation from the respiratory mucosa, increasing mucus viscosity, reducing ciliary motility efficiency by over 50%, and weakening pathogen clearance capacity (51, 52).

**Figure 2:**
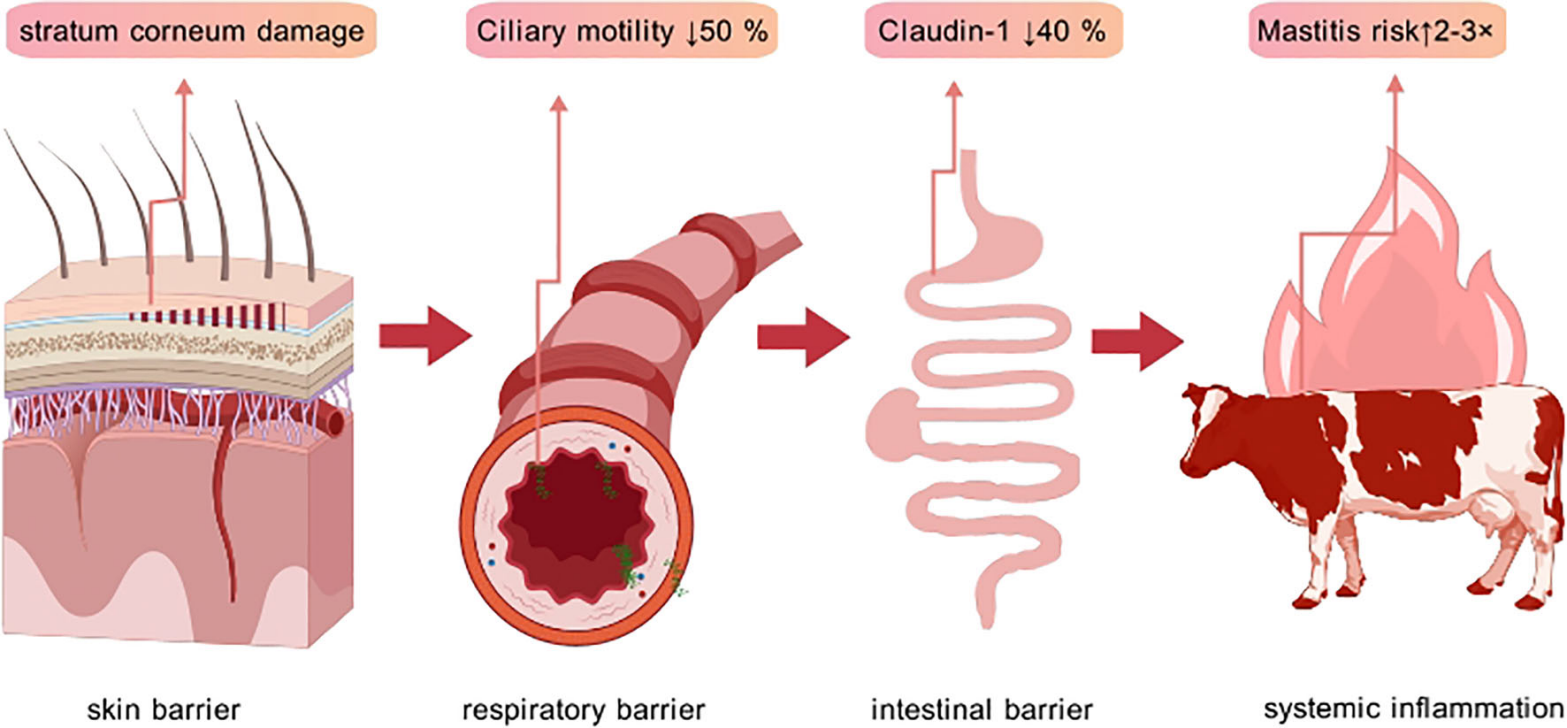
The multi-level disruption of the innate immune barrier GDP. Heat stress disrupts the three primary innate immune barriers, promoting endotoxin translocation and precipitating a systemic inflammatory storm.

The intestinal barrier is a key component of the bovine immune barrier impacted by heat stress. Heat stress can downregulate the expression of tight junction proteins (e.g., Claudin-1, Occludin) in intestinal epithelial cells, impairing barrier function and increasing intestinal permeability (53). This barrier dysfunction allows antigens like bacteria and toxins from the gut lumen to more easily enter the bloodstream, triggering systemic immune responses (**Figure 2**) (54). For example, heat stress downregulates Claudin-1 and Occludin expression, increasing paracellular permeability and widening intestinal barrier “leaks” (55). Heat stress may also alter the structure and function of the gut microbiota, leading to a reduction in beneficial bacteria and an increase in harmful bacteria, thereby disrupting gut microbial balance (56). For instance, increased intestinal permeability allows endotoxin (LPS) and Gram-negative bacteria to translocate into the circulation, elevating serum LPS concentrations and triggering systemic inflammation (57). Gut dysbiosis not only affects digestive and absorptive functions but may also reduce disease resistance by impacting the development and function of the gut mucosal immune system (58). For example, the abundance of beneficial bacteria (e.g., Lactobacillus) in the gut decreases by ~60%, while conditional pathogens (E. coli) proliferate 2.8-fold, exacerbating intestinal inflammation (59–61). Dysbiosis leads to decreased production of short-chain fatty acids (SCFAs, e.g., butyrate), weakening their role in energy supply and anti-inflammatory regulation for gut epithelial cells (62).

### 3.2 Impact of heat stress on bovine innate immune factors

Heat stress impairs innate immune function through multiple avenues. It significantly compromises the function of innate immune cells, including the phagocytic capacity and chemotaxis of macrophages, monocytes, and neutrophils, reducing the cow’s pathogen clearance ability and the speed of local immune responses (63). Studies show heat stress alters cytokine levels: pro-inflammatory cytokines (e.g., TNF-α, IL-6) increase, while changes in immunosuppressive factors (e.g., IL-10) can lead to immune system imbalance, placing cows in a suboptimal health state (64, 65). Heat stress also suppresses the synthesis and secretion of antimicrobial peptides (e.g., defensins, cathelicidins), reducing the cow’s direct defense capability against pathogens (24). Research indicates a decline in the opsonophagocytic index (OPI), reduced efficiency of membrane attack complex (MAC) formation, and prolonged clearance cycles for Gram-negative bacteria (66, 67).

Regarding blood immune parameters, heat stress causes a decrease in leukocyte counts, particularly lymphocytes and neutrophils, while also affecting the synthesis and secretion of immunoglobulins (e.g., IgG, IgM), further weakening humoral immune responses (68). Heat stress upregulates the glucocorticoid-induced protein GILZ, suppressing neutrophil chemotactic activity, and excessive ROS reduces phagocytic capacity by ~50% (69, 70). M2 polarization of macrophages is hindered, IL-10 secretion decreases, while TNF-α secretion increases, exacerbating tissue oxidative damage (71–73).

Oxidative stress is another key mechanism of heat stress impact. Heat stress increases free radical generation, causing oxidative damage that directly harms cells and impairs immune cell function, reducing the synthesis of immune factors (74). Chronic heat stress may lead to reduced immune tolerance, increasing the cow’s susceptibility to infections like mastitis and respiratory diseases (75). Concerning mammary immunity, heat stress impairs the function of mammary leukocytes, reduces concentrations of innate immune factors like whey immunoglobulins and lysozyme, and weakens the innate immune defense capacity of the mammary gland (76).

In summary, heat stress significantly impairs the phagocytic capacity and chemotaxis of macrophages, monocytes, and neutrophils, reducing the cow’s pathogen clearance ability and the speed of local immune responses. Concurrently, heat stress alters cytokine profiles: increased pro-inflammatory cytokines (e.g., TNF-α, IL-6) and altered immunosuppressive factors (e.g., IL-10) may cause immune system imbalance, placing cows in a suboptimal health state. Additionally, heat stress suppresses the synthesis and secretion of antimicrobial peptides (e.g., defensins, cathelicidins), reducing the cow’s direct defense against pathogens. Heat stress causes a decline in the opsonophagocytic index (OPI), reduced membrane attack complex (MAC) formation efficiency, and prolonged clearance cycles for Gram-negative bacteria. Blood immune parameters show decreased leukocyte counts, particularly lymphocytes and neutrophils, alongside impaired immunoglobulin (e.g., IgG, IgM) synthesis and secretion, further weakening humoral immunity. Oxidative stress markers increase; heat stress elevates free radical generation, causing oxidative damage that directly harms cells and impairs immune cell function, reducing immune factor synthesis. In mammary immunity, heat stress impairs mammary leukocyte function, reduces concentrations of innate immune factors like whey immunoglobulins and lysozyme, and weakens mammary innate defense capacity. Heat stress impairs bovine innate immune function through multiple mechanisms; these changes serve as potential biomarkers for monitoring and assessing the impact of heat stress on cow health.

## 4 Impact of heat stress on bovine adaptive immunity

Heat stress exerts significant negative effects on the bovine adaptive immune system, primarily manifesting as suppression of immune cell function, imbalance in immune factor expression, and enhanced immune tolerance (77). Regarding immune cells, heat stress markedly inhibits the proliferation and differentiation capacity of T cells and B cells; specifically, CD4+ T cell proliferation declines, thymocyte apoptosis increases, leading to significantly weakened cytotoxic responses (78). B cell antibody affinity maturation is impaired, activation-induced cytidine deaminase (AID) expression decreases, serum IgG titers fall, and the efficiency of immune memory formation post-vaccination is reduced (79).

Adaptive immunity, the core defense mechanism for specific pathogen recognition and clearance, shows significant functional suppression under heat stress (80). Bovine adaptive immunity, mediated by T and B lymphocytes, exhibits acquired characteristics, memory, and high specificity, playing a key role in maintaining immune homeostasis (81). Heat stress disrupts the functional balance of this system through multiple pathways. For T cells, thymic microenvironment disturbances lead to decreased CD4^+^ T cell proliferation capacity, Th1/Th2 cytokine imbalance, and significantly weakened cytotoxic responses (82). B cells exhibit impaired antibody affinity maturation, reduced serum IgG titers, and decreased efficiency of immune memory formation post-vaccination (83). Heat stress-induced elevation of glucocorticoid levels suppresses the NF-κB signaling pathway, downregulating macrophage phagocytic activity and natural killer (NK) cell cytotoxicity, creating a state of systemic immunosuppression (84). Chronic heat stress can further lead to enhanced immune tolerance, increasing the risk of mastitis and respiratory diseases. Studies show that selenium and vitamin E supplementation can partially reverse heat stress damage (85).

### 4.1 Impact of heat stress on bovine cell-mediated immunity

Heat stress is a common physiological stress response in cows exposed to high temperatures, significantly impacting their immune system, particularly cell-mediated immunity. Heat stress disrupts immune cell function and immune factor balance through multiple mechanisms, thereby weakening the cow’s disease resistance. Heat stress alters immune cell function and distribution. Changes in peripheral blood leukocyte distribution manifest as increased neutrophil counts and decreased lymphocyte counts; this shift may promote chronic inflammation and weaken disease resistance (86, 87). Macrophage phagocytosis, antigen presentation, and cytokine secretion functions are suppressed, reducing immune response capacity (88). The proliferation and differentiation capacities of T cells and B cells are also inhibited, further impairing adaptive immune responses (89, 90).

Heat stress reprograms the bovine immune system through multiple pathways. When exposed to high temperatures, the hypothalamic-pituitary-adrenal (HPA) axis is rapidly activated, leading to a significant rise in glucocorticoid levels (primarily cortisol) (91). Cortisol, a core stress hormone, binds to glucocorticoid receptors (GRs) on immune cells, suppressing the transcription of pro-inflammatory cytokines (e.g., IL-2, IFN-γ) while inducing lymphocyte apoptosis, ultimately establishing a state of immunosuppression (92). Research confirms that serum cortisol levels in heat-stressed cows can be 2–3 times higher than in non-stressed periods, showing a significant negative correlation with decreased total leukocyte count and reduced neutrophil phagocytic function (93).

Simultaneously, heat stress induces mitochondrial dysfunction, prompting excessive accumulation of reactive oxygen species (ROS) and triggering oxidative stress (94). This directly causes protein denaturation, lipid peroxidation, and DNA damage, accompanied by the collapse of the antioxidant enzyme system—superoxide dismutase (SOD) and glutathione peroxidase (GSH-Px) activity significantly decrease, while levels of the oxidative end-product malondialdehyde (MDA) significantly increase (95, 96). Studies show SOD concentration is significantly negatively correlated with THI (r=-0.406), while MDA content is significantly positively correlated with THI (r=0.381), highlighting the close link between oxidative stress and heat load (97).

Heat stress also affects immune cell function via metabolic reprogramming. For example, key metabolites like linoleic acid and fructose are downregulated during heat stress; these molecules are not only energy substrates but also immunoregulatory signaling molecules (98). Metabolic disturbances form a vicious cycle with gut dysbiosis (e.g., reduced Prevotella), further impairing intestinal barrier function, increasing endotoxin translocation risk, and ultimately leading to systemic inflammation (99, 100).

The Neutrophil-to-Lymphocyte Ratio (NLR), an indicator of systemic inflammation, significantly increases during heat stress (101). Neutrophil numbers decrease, and their chemotaxis and phagocytic function are impaired, while lymphocyte subset ratios become imbalanced: the CD4+/CD8+ T cell ratio decreases, and B cell antibody production capacity is reduced (102). Research indicates lymphocyte counts in heat-stressed cows are significantly lower than in non-stressed groups (P<0.05), while levels of pro-inflammatory cytokines like IL-2, IL-6, and TNF-α are significantly elevated (P<0.05) (103).

A shift in monocyte functional phenotype is also significant. Heat stress induces monocyte differentiation towards anti-inflammatory M2-type macrophages, highly expressing arginase 1 (Arg1) and IL-10, thereby suppressing Th1-type immune responses (104). While this phenotypic shift helps limit inflammatory damage, it concurrently weakens the body’s ability to clear pathogens, increasing the risk of mastitis and metritis.

A burst increase in pro-inflammatory cytokines is a core feature of the early pathophysiology of heat stress in cows. Research finds plasma levels of IL-6, TNF-α, and IL-1β are significantly elevated in heat-stressed cows, with TNF-α showing a significant positive correlation with the THI index (r=0.423) (103, 105). These cytokines amplify inflammatory cascades by activating signaling pathways like NF-κB and mediate typical symptoms such as fever and anorexia (106). Notably, IL-6 plays a dual role: as a pro-inflammatory cytokine in the acute phase response, and by inhibiting thyroid hormone secretion to reduce heat production, forming an adaptive feedback loop (107, 108).

**Figure 3:**
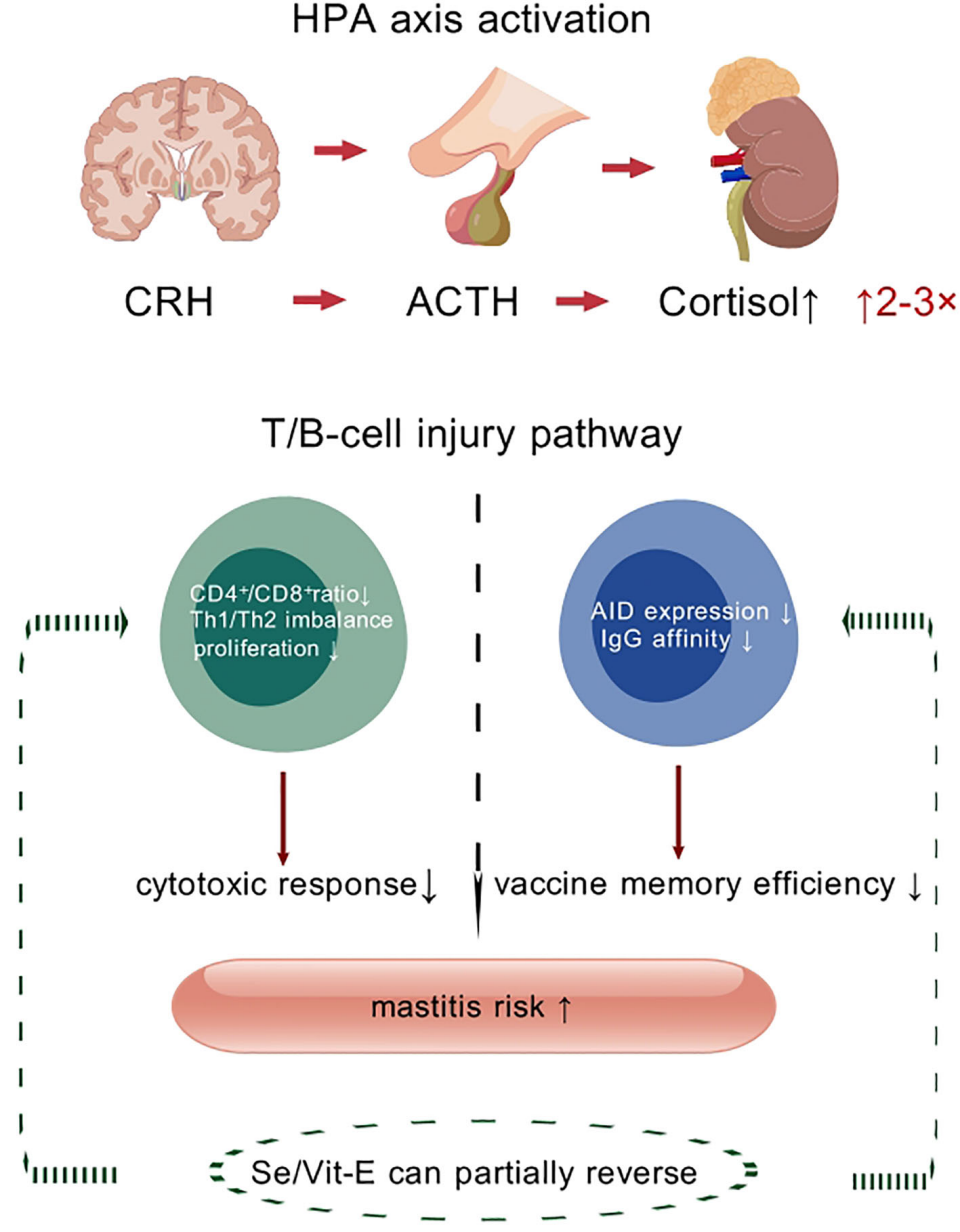
Adaptive immune suppression and memory impairment GDP. Heat stress suppresses T- and B-cell functionality via the HPA axis–glucocorticoid pathway, thereby attenuating vaccine-mediated protection.

Acute-phase proteins (APPs) like haptoglobin (Hp) and serum amyloid A (SAA) rise significantly during heat stress, serving as sensitive indicators of tissue damage (109, 110). They are primarily synthesized by the liver in response to inflammatory signals and participate in processes like opsonization and free radical scavenging (111). Studies show Hp levels in heat-stressed cows correlate positively with rectal temperature (RT), suggesting their potential as markers for assessing inflammatory load (112, 113) (**Figure 3**).

### 4.2 Impact of heat stress on bovine humoral immunity

Innate immune factors rapidly recognize and respond to foreign pathogens. In high-temperature environments, the cow’s humoral immune system is significantly affected. Heat stress suppresses the function of key immune cells like T cells, B cells, and macrophages. T cell numbers decrease, proliferation declines, weakening cell-mediated immune responses (114); B cell antibody synthesis and secretion capacity is impaired, affecting immune memory establishment (115); macrophage phagocytic ability and inflammatory cytokine generation are also suppressed, reducing the overall defensive capacity of the immune system (116). Heat stress significantly impacts antibody production in cows, particularly the synthesis and secretion of immunoglobulins like IgG, IgA, and IgM (117). IgG is the most important antibody in milk; its decreased level weakens immune protection for newborn calves (118). Reduced antibody production is closely linked to endocrine changes induced by heat stress (e.g., elevated cortisol). Cortisol suppresses B and T cell function, further impacting antibody production (119). Heat stress triggers a series of inflammatory reactions, elevating levels of cytokines like IL-1β, IL-6, and TNF-α (120). While these cytokines help initiate acute immune responses, chronic inflammation can lead to immunosuppression and disrupt immune system balance (121). Oxidative stress induced by heat stress increases free radical generation, damaging immune cell function (122). Heat stress also affects mammary blood circulation in cows, reducing the delivery of immune cells and transport of antibodies, thereby impacting the levels of immune components in milk (123). Decreased levels of IgG and IgA in milk weaken immune protection for newborn calves (124). Heat stress also increases the risk of mammary inflammation, further affecting milk quality and the cow’s immune status (125). Heat stress causes elevated levels of stress hormones like cortisol (126). Cortisol has immunosuppressive effects, reducing immune cell proliferation and cytokine secretion, thereby suppressing normal immune system function (127). Chronically elevated cortisol levels are a major contributor to reduced immunity in cows (128). Heat stress impairs the function of mammary immune cells (e.g., macrophages, lymphocytes), reduces the mammary gland’s ability to secrete immune factors, and increases the risk of mammary infection (129). Chronic heat stress may also reduce the number of local mammary immune cells, further impacting milk immunity (130).

## 5 Impact of heat stress on mammary system immunity

Heat stress exerts multifaceted effects on the mammary system immunity of milk-producing animals like dairy cows. It causes a reduction in immune function, weakening the animal’s defense against pathogens, increasing the risk of udder infection by pathogenic bacteria, and thereby predisposing to mastitis. Studies indicate that under heat stress conditions, immunoglobulin concentrations decrease and cytokine levels increase in cow blood, suppressing overall immune function (131). At the cellular level, heat stress alters the composition and activity of immune cells within the mammary gland. Lengi et al. (2022), using flow cytometry analysis, found that compared to cows under thermoneutral conditions, heat-stressed cows had significantly elevated concentrations of mammary epithelial cells in milk, while viable granulocytes and total viable CD45^+^ cells decreased by 17% and 12%, respectively. This indicates heat stress affects the relative numbers and viability of certain somatic cell populations in milk; reduced immune cell viability may negatively impact mammary immune capacity in cows (15). Heat stress also interferes with the normal physiological functions and defense mechanisms of the mammary gland. It induces physiological and metabolic disturbances in cows, including altered blood circulation and endocrine dysregulation; these changes may impair normal mammary function, creating favorable conditions for pathogen invasion and proliferation (132). For example, reduced feed intake during heat stress leads to insufficient nutrient intake, affecting systemic immunity and defense; excessive water intake dilutes antimicrobial components in milk, reducing the udder’s ability to resist infection (133). During the dry period, heat stress may negatively impact mammary involution due to endocrine changes (decreased estrogen, increased prolactin). Immune cells play a crucial role in preparing mammary tissue for the next lactation during this stage; heat stress disruption of this process may impair immune protection in the subsequent lactation (134). Under heat stress, milk retention and reduced flow within the udder favor pathogen colonization and proliferation in the mammary gland, thereby inducing mastitis (135). Research indicates that heat load significantly influences the occurrence of clinical mastitis in dairy cows; higher milk yield, later lactation stage, and greater parity increase the risk of clinical mastitis under heat stress (136).

### 5.1 Impact of heat stress on bovine mammary immune homeostasis

Heat Stress is a significant environmental stress factor in modern dairy farming, adversely affecting cow productivity, mammary immune function, and milk quality (137). The impact of heat stress on the bovine immune system is multifaceted, particularly concerning mammary immune homeostasis (138). Immune system dysregulation can lead to mammary infections, subsequently affecting milk quality (27). Heat stress alters immune cell function, including reduced numbers or impaired function of peripheral blood leukocytes, and weakened phagocytosis by macrophages and dendritic cells, increasing mastitis risk (139). Heat stress also leads to enhanced inflammatory responses and elevated levels of stress hormones (e.g., cortisol, epinephrine) (140, 141), suppressing normal immune cell function, especially anti-infective capacity (142). Chronic heat stress may induce chronic low-grade systemic inflammation; local mammary inflammatory responses are consequently heightened, predisposing to immune-related diseases like mastitis (143). Heat stress also reduces the secretion of immunoglobulins (e.g., IgG, IgA), weakening local mammary immune defense and making cows more susceptible to pathogenic microorganisms (144). Cytokine imbalance is another significant effect of heat stress: pro-inflammatory cytokine levels (e.g., TNF-α, IL-1β, IL-6) rise (145), while anti-inflammatory cytokine levels (e.g., IL-10) fall, promoting inflammation onset and exacerbating immunosuppression (146).

Specific impacts of heat stress on mammary health include increased mastitis incidence, mammary microbial dysbiosis, and reduced milk quality (147). Mastitis is a common mammary infectious disease, typically caused by bacteria (148, 149). Heat stress-induced decline in immune system function weakens mammary defense against pathogens, thereby increasing mastitis incidence (150). The mammary gland harbors a microbial community that plays a role in maintaining immune homeostasis (151). Heat stress may alter the normal mammary microbiota (152), increasing the proliferation of harmful bacteria (e.g., Staphylococcus aureus, Streptococcus spp.), leading to mammary infection (153). Due to impaired mammary immune function, cows under heat stress may secrete more immune-related molecules (e.g., lactoferrin, enzymes), which can affect milk composition, leading to reduced milk quality (44). For example, milk fat and protein content may decrease (154), while levels of casein, lactose, etc., may change, affecting final dairy product quality (155).

The impact of heat stress on bovine mammary immune homeostasis is multifaceted. By reducing the defensive capacity of the immune system, exacerbating inflammatory responses, and altering the mammary microbiota, it contributes to mammary health problems and affects dairy product quality (156, 157). Improving the environment, optimizing management, and enhancing immunity can effectively mitigate the negative impacts of heat stress, ensuring cow productivity and milk quality.

### 5.2 Impact of heat stress on bovine mastitis

The impact of heat stress on bovine mastitis is a complex, multi-dimensional issue. Particularly under hot and humid conditions, heat stress significantly affects the cow’s physiological state, immune system, and mammary health, thereby adversely influencing mastitis incidence, severity, and treatment efficacy (158). Heat stress causes elevated body temperature and increased respiratory rate; these physiological changes not only impair immune cell function but also reduce the defensive capacity of skin and mammary tissues, increasing the risk of pathogen invasion (159). Research shows that under heat stress, the somatic cell count (SCC) in cow milk significantly increases, directly correlating with mastitis incidence (136). Due to immune system impairment by heat stress, the mammary defense capacity is significantly reduced when encountering bacterial infections, leading to a marked rise in mastitis incidence (160). Studies demonstrate a clear correlation between heat stress and mastitis, especially during summer or hot, humid seasons, when the risk of mastitis outbreaks increases (161). Heat stress not only aggravates clinical mastitis but also worsens subclinical mastitis symptoms, causing chronic inflammation to progress to clinical mastitis (160). Heat stress may also affect milk quality, manifesting as increased udder temperature, reduced milk yield, and altered milk composition (162). Mastitis itself elevates mammary tissue temperature, further impacting milk quality (163). Heat stress negatively impacts the cow’s overall physiological state, leading to reduced milk production (164). Mastitis and heat stress may also increase bacterial content in milk, leading to decreased protein, fat, and lactose levels, affecting dairy product quality (165).

Heat stress not only weakens the cow’s immune system but also creates favorable conditions for pathogen growth and dissemination. It increases free radical generation and decreases antioxidant enzyme activity (e.g., SOD, GSH-Px); oxidative damage impairs immune cell function (166). This oxidative stress state directly damages cells and disrupts immune-metabolic processes, reducing immune cell proliferation and differentiation capacity (167, 168).

**Figure 4:**
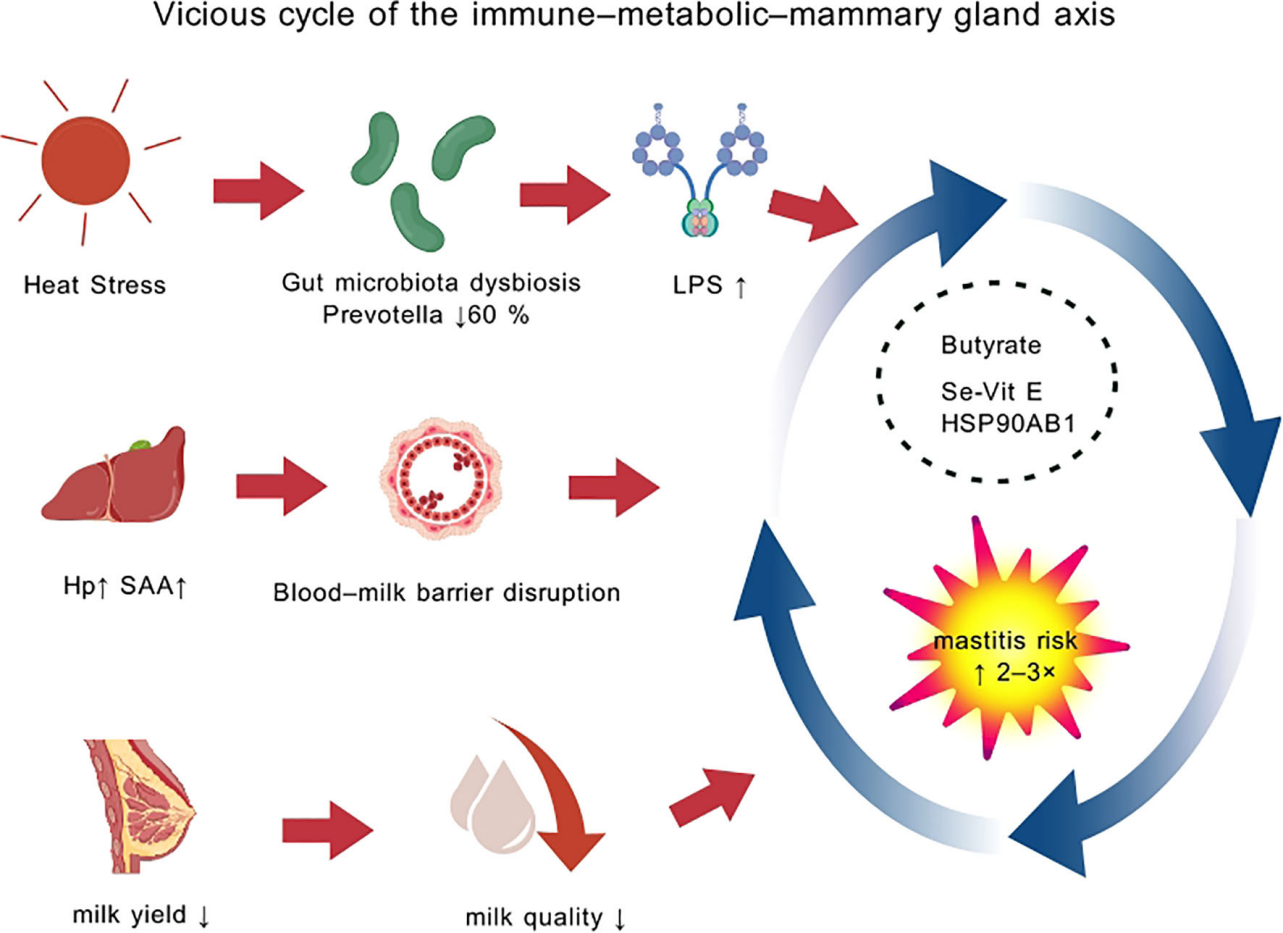
Vicious cycle of the immune–metabolic–mammary axis. Heat stress drives a vicious gut–liver–mammary axis and identifies potential intervention targets.

. Heat stress damages tight junction proteins (e.g., Claudin-3, Occludin) in mammary epithelial cells, increasing paracellular permeability and impairing blood-milk barrier function (169) (**Figure 4**). This barrier disruption allows pathogens and inflammatory factors to penetrate mammary tissue more easily, significantly increasing infection risk (170). Heat stress increases serum levels of pro-inflammatory cytokines (e.g., TNF-α, IL-6) while decreasing anti-inflammatory cytokines (e.g., IL-10), causing immune system imbalance; this inflammatory dysregulation further affects metabolic processes, creating a vicious cycle (171). Under heat stress, the disruption of mammary epithelial tight junctions, reduced concentrations of antimicrobial substances like lysozyme and lactoferrin in milk, and increased colonization by pathogens like S. aureus occur (172). Elevated levels of IL-8 and TNF-α in milk trigger excessive inflammatory responses, while insufficient anti-inflammatory factors exacerbate mammary tissue damage, potentially increasing clinical mastitis incidence by 2–3 fold (173, 174). Heat stress significantly increases mastitis incidence in dairy cows through multiple mechanisms: weakening the immune system, disrupting mammary barrier function, and exacerbating inflammation.

To mitigate the negative impact of heat stress on bovine mastitis, farm managers need to implement measures including environmental control, water management, nutritional modulation, enhanced udder care, and regular health monitoring. These strategies help improve cow living conditions and immune status, effectively reducing heat stress-related mastitis risk and enhancing production efficiency and milk quality (175). The impact of heat stress on bovine mastitis is multifaceted, involving both decreased immune system function and compromised mammary health (176). To reduce its negative effects, adopting scientific environmental control, nutritional supplementation, and mammary health management is crucial. Optimizing management and preventive measures can effectively lower heat stress-induced mastitis risk and improve cow productivity and milk quality.

## 6 Impact of heat stress on bovine immunometabolism

Heat stress profoundly impacts bovine immunometabolism, involving multifaceted changes in immune cell function, oxidative stress, endocrine regulation, and overall metabolic status. At the immune cell level, heat stress significantly suppresses neutrophil chemotaxis and bactericidal capacity, reduces macrophage phagocytic activity, and impairs lymphocyte proliferation and differentiation (177). These changes weaken the cow’s pathogen clearance ability, increasing infection risk. Increased oxidative stress is another key mechanism, characterized by excessive generation of reactive oxygen species (ROS) and reactive nitrogen species (RNS), exceeding the clearance capacity of antioxidant systems, leading to cellular damage and further suppression of immune cell function (178). Regarding endocrine regulation, heat stress activates the hypothalamic-pituitary-adrenal (HPA) axis, elevating cortisol levels, which subsequently suppresses immune cell activity and cytokine secretion (179). Heat stress also alters thyroid hormone levels, reducing basal metabolic rate and energy supply for immune cells. At the systemic metabolic level, heat stress causes negative energy balance, lowering serum glucose and non-esterified fatty acid (NEFA) levels while increasing lactate accumulation, impairing normal immune system function (180). These combined effects not only weaken the cow’s immune defense but also significantly negatively impact production performance and health (181).

### 6.1 Overview of bovine immunometabolism

Bovine immunometabolism refers to the metabolic processes involved in the immune system maintaining normal physiological function and responding to pathogen invasion (182). It encompasses not only energy metabolism and nutrient allocation but also involves oxidative stress, endocrine regulation, and the functional state of immune cells (183). Proper functioning of immunometabolism is crucial for maintaining cow health and productivity. At rest, immune cells rely on highly energy-efficient processes like the tricarboxylic acid (TCA) cycle, while activated immune cells shift towards glycolysis. This metabolic reprogramming not only affects immune cell function but may also influence systemic metabolism through metabolites and signaling molecules (184–186).

The periparturient period is a critical phase for immunometabolic changes in cows. During this stage, cows undergo physiological processes like placental expulsion, uterine involution, fat mobilization, and lactogenesis, all accompanied by tissue remodeling and inflammatory responses (187). Fat mobilization promotes inflammatory responses in adipose tissue, releasing large amounts of non-esterified fatty acids (NEFA), thereby affecting systemic metabolism (188, 189). Circulating glucose in postpartum cows is preferentially allocated to immune cells and the mammary gland; the activated immune system further consumes glucose, exacerbating negative energy balance (190). Immunometabolism is closely linked to bovine health. Mastitis is a common immunometabolic disease; its major pathogen, Staphylococcus aureus (SA), evades host immunity by secreting metabolic and virulence factors like lipases (191–193). Periparturient cows often experience metabolic inflammation, characterized by elevated serum levels of pro-inflammatory cytokines (e.g., IL-6, TNF-α) and acute-phase proteins (e.g., Serum Amyloid A, SAA); this inflammatory state affects the appetite center, leading to hypoglycemia, hypoinsulinemia, and reduced feed intake, further worsening negative energy balance (194, 195). Immunometabolic regulation can be achieved through nutritional and immunological interventions. For example, Niacin, a known inhibitor of fat mobilization, can reduce fat mobilization during the transition period, alleviating metabolic inflammation, while also influencing immune cell metabolic state, promoting a shift from pro-inflammatory to anti-inflammatory phenotypes (196–198). By reducing inflammatory factor production, fat mobilization and hepatic metabolic disturbances can be mitigated (199).

Bovine immunometabolism exhibits significant dynamic changes during the periparturient period; its dysregulation is closely associated with various diseases. Nutritional and immunomodulatory approaches can effectively improve metabolic and immune status, providing a theoretical basis for healthy dairy farming.

### 6.2 Immunometabolic biomarkers in the heat stress environment

Heat stress increases free radical generation in cows and decreases the activity of antioxidant enzymes like superoxide dismutase (SOD) and glutathione peroxidase (GSH-Px); oxidative damage impairs immune cell function (200). This oxidative stress state directly damages cells and disrupts immunometabolic processes, reducing immune cell proliferation and differentiation capacity (201). Heat stress reduces feed intake, decreasing energy intake, while the energy required to maintain body temperature increases, leading to negative energy balance (202). This shift in energy metabolism affects immune system function, as immune responses demand substantial energy support. Studies show heat stress lowers serum glucose and non-esterified fatty acid (NEFA) levels while increasing lactate accumulation (203). Heat stress significantly impacts immune cell function, including neutrophils, macrophages, and lymphocytes. Research indicates it suppresses neutrophil chemotaxis and bactericidal capacity, reduces macrophage phagocytic activity, and impairs lymphocyte proliferation and differentiation (63, 74). Heat stress also increases immune cell apoptosis, further reducing immune system function (204). Heat stress activates the hypothalamic-pituitary-adrenal (HPA) axis, elevating cortisol levels (205, 206). Cortisol has immunosuppressive effects, reducing immune cell proliferation and cytokine secretion (207). Heat Shock Proteins (HSPs) are among the most important molecules in the heat stress response; they not only exert protective effects during cellular damage but also mitigate cell injury and enhance immune defense against pathogens by regulating protein folding, antioxidant responses, and immune reactions (208). Heat stress also suppresses thyroid function, reducing thyroid hormone (T3, T4) levels, lowering basal metabolic rate and energy supply for immune cells (209). Heat stress affects not only local immunometabolism but also systemic immunometabolism through multiple mechanisms. Studies show it increases serum levels of pro-inflammatory cytokines (e.g., TNF-α, IL-6) while decreasing anti-inflammatory cytokines (e.g., IL-10), causing immune system imbalance (145). This immune dysregulation further impacts metabolic processes, creating a vicious cycle. Heat stress also affects gut microbiota structure, alters intestinal barrier function, increases the risk of endotoxemia, and further disrupts systemic immunometabolism (210, 211).

Heat stress causes significant alterations in the plasma metabolome. Targeted metabolomic analysis reveals that 9 metabolites, including linoleic acid and fructose, are downregulated during heat stress; these molecules are not only energy sources but also signaling molecules regulating immune cell function (2, 212). Linoleic acid, a polyunsaturated fatty acid, participates in synthesizing anti-inflammatory mediators like resolvins; decreased fructose levels are directly linked to gut dysbiosis, affecting short-chain fatty acid production (213).

The gut microbiota-immune axis plays a key role in heat stress. Genus-level analysis shows a significant decrease in Prevotella abundance in the feces of heat-stressed cows; this genus is a major player in dietary fiber fermentation and butyrate production (214). Butyrate, a primary energy source for colonocytes, has multiple functions: maintaining intestinal barrier integrity, inducing regulatory T cell differentiation, and inhibiting NF-κB activity (215). Its reduction leads to increased intestinal permeability, endotoxin translocation, and triggers systemic low-grade inflammation. Conversely, the relative abundance of opportunistic pathogens like Escherichia increases, further elevating infection risk (216).

## 7 Discussion

Heat stress impacts the bovine immune system multifariously, involving barrier function disruption, altered immune cell activity, and dysregulation of the metabolism-immune axis. Significant downregulation of intestinal tight junction proteins (Claudin-1/Occludin) causes endotoxin translocation, triggering systemic inflammation and disrupting the blood-milk barrier, markedly increasing mastitis risk. Reduced efficiency of respiratory mucosal ciliary movement also impairs pathogen clearance. Regarding immune cell function, decreased neutrophil chemotaxis and macrophage phagocytic activity, coupled with suppressed antimicrobial peptide synthesis, weaken innate immunity. Adaptive immunity is characterized by a reduced CD4+/CD8+ T cell ratio, impaired B cell antibody affinity maturation, and diminished vaccine-induced immune memory efficiency. Dysregulation of the metabolism-immune axis further exacerbates the problem; heat stress-induced metabolic reprogramming and gut dysbiosis form a vicious cycle, leading to heightened oxidative stress. These findings reveal the systemic impact of heat stress on bovine health, providing a crucial foundation for subsequent research.

The immune-related biomarkers proposed in this study demonstrate significant potential for early warning, individualized assessment, and breeding guidance. Changes in the Neutrophil-to-Lymphocyte Ratio (NLR), Heat Shock Proteins (HSP70/HSP90), and acute-phase proteins (Haptoglobin, Hp) precede declines in production performance, providing a basis for early heat stress intervention. Furthermore, high-yielding cows exhibit metabolic disturbances at a lower Temperature-Humidity Index (THI = 65), which traditional THI thresholds (>68) fail to capture, highlighting the value of novel biomarkers for individualized assessment. For breeding, the CD4+/CD8+ T cell ratio and HSP gene polymorphisms can serve as molecular markers for thermotolerance traits, accelerating the selection of heat-tolerant lineages and supporting the sustainable development of the dairy industry.

Despite the progress made, several unresolved questions and research bottlenecks remain. Firstly, the precise mechanisms by which heat stress regulates epigenetic markers (e.g., DNA methylation) to influence immune gene expression are unclear and require further elucidation using multi-omics technologies. Secondly, detection thresholds for cytokine profiles (e.g., IL-6/TNF-α) are not standardized, and the lack of rapid, on-farm detection systems limits the widespread application of novel biomarkers. Additionally, current intervention strategies lack sufficient timeliness; for instance, selenium/vitamin E supplementation only partially reverses heat stress damage. Future research needs to develop targeted interventions focusing on the gut-mammary axis (e.g., butyrate formulations) to improve efficacy.

Based on these findings, this study proposes several practical recommendations. Firstly, integrating the Temperature-Humidity Index (THI) with immune biomarkers (e.g., NLR, HSP70) to construct a dynamic risk assessment model can enable individualized monitoring of cow heat stress, enhancing the precision of early warning systems. Secondly, optimizing nutritional intervention strategies, such as adding butyrate precursors (e.g., dietary fiber), can help maintain gut microbial balance, alleviate endotoxin translocation, and mitigate the negative health impacts of heat stress. Furthermore, incorporating indicators like HSP90AB1 gene polymorphism and CD4+ T cell activity into genomic selection indices holds promise for accelerating the breeding of heat-tolerant dairy cattle lines. For mammary health management, strengthening heat stress mitigation measures (e.g., cooling facilities) during the dry period can reduce the risk of abnormal mammary epithelial cell proliferation and safeguard udder health.

Against the backdrop of global warming, heat stress has become a major challenge for the dairy industry. The application of immune biomarkers offers a new paradigm shifting from “passive response” to “active prevention and control” in heat stress management. Future research requires deep integration of immunology, metabolomics, and epigenetics to construct cross-scale regulatory networks, driving the upgrade of climate resilience in dairy farming and providing solid scientific and technical support for the industry’s sustainable development.
